# Interventions Aimed at the Prevention of Childhood Injuries in the Indigenous Populations in Canada, Australia and New Zealand in the Last 20 Years: A Systematic Review

**DOI:** 10.3390/ijerph14060589

**Published:** 2017-06-02

**Authors:** Alyssa Margeson, Selena Gray

**Affiliations:** Department of Health and Social Sciences, University of the West of England, Coldharbour Lane, Bristol BS16 1QY, UK; selena.gray@uwe.ac.uk

**Keywords:** systematic review, interventions, aboriginal, Indigenous, injury, childhood, evaluation, prevention, strategy, public health

## Abstract

Globally, Indigenous children are found to be at a significantly higher risk of injury compared to non-Indigenous children. It has been suggested that mainstream injury prevention strategies are ineffective within Indigenous communities. The aim of this review is to identify existing interventions aimed at preventing injury in Indigenous children in the hope that it guides future strategies. To the best of the authors’ knowledge, no prior systematic reviews exist looking at interventions specifically aimed at preventing injury in Indigenous child populations in the three chosen countries. Electronic databases were systematically searched for relevant childhood interventions aimed at the prevention of injuries in Indigenous populations based in Canada, Australia and New Zealand from 1996 to 2016. A manual search of the reference lists of relevant articles and a manual search of relevant websites were also completed. After 191 records were screened, six interventions were identified meeting the criteria for inclusion. Eligible papers underwent a quality appraisal using adapted assessment checklists and key information was extracted. Findings were then synthesized using a narrative approach. The interventions mainly promoted child safety through activities focusing on education and awareness. Only three of the six studies measured changes in injury hospitalization rates, all but one evaluation reporting a significant decrease. Studies which measured awareness all demonstrated positive changes. Results suggest that interventions delivered in a culturally appropriate manner acted as a main success factor. Barriers identified as hindering intervention success included lack of cohesion within the intervention due to staff turnover and lack of experienced staff with Indigenous knowledge. This review revealed a limited amount of evaluated interventions for the prevention of Indigenous childhood injuries. Conclusive evidence of the effectiveness of existing interventions is lacking due to the predominantly small-scale evaluations of pilot interventions. Future research is needed to provide more rigorous evidence of the mechanisms driving the successful implementation, delivery and uptake of such strategies tailored to Indigenous children.

## 1. Introduction

Canada, Australia and New Zealand are among the top developed countries in the world with the largest percentages of Indigenous populations [1]. The *Human Development Index* (*HDI*) developed by the United Nations Development Programme (UNDP) measures average achievement in three basic dimensions of human development: a long and healthy life, knowledge, and a decent standard of living [2]. Canada, Australia and New Zealand consistently are ranked high on this index; Canada ranking 10th, Australia ranking 2nd and New Zealand ranking 13th, out of a total of 188 countries evaluated in 2015 [2]. These countries are prided on their health, economic development and universal healthcare access but despite this, the overall health of Indigenous populations is poor compared to the non-Indigenous individuals residing in these countries [1].

Globally, by the year 2020, it is projected that injury will cause 8.4 million deaths annually [3]. More concerning is the unfortunate fact that these deaths are shown to be unevenly distributed among Indigenous populations worldwide [4,5]. Injury is a major cause of mortality and disability among Indigenous children in these countries [6,7,8]. Indigenous children in Canada, Australia and New Zealand are found to be at a significantly higher risk of injury [9,10,11]. A systematic review looking at unintentional child injury rates globally found that the morbidity rate ratio for Indigenous to non-Indigenous ranged from 1.2 to 2.3 [9]. Transport injuries, in particular, were found to demonstrate the greatest inequality in younger age groups across different communities worldwide [9]. In areas of high-percentage Canadian First Nations identity areas, the age-standardized unintentional injury hospitalization rate for 0–19 years old was found to be 85.9 per 10,000, compared to 37.1 in areas of low-percentage Aboriginal identity areas [12].

The explanation for the overall observed health disparities and higher injury rates in Indigenous populations in these countries is complex. Past colonization has caused the removal of Indigenous people from their land which has subsequently impacted their cultural and social well-being [5]. Canada, Australia and New Zealand have similar colonial histories [4] marked with discrimination and oppression [13]. These effects of colonialism remain impactful in present generations [14] and the current socioeconomic disadvantage experienced by Indigenous populations has resulted in exposure to behavioral and environmental health risks [15]. Consequently, high unemployment rates, remoteness, unideal living conditions and a lack of education are factors contributing to injury [15].

The reason for the persistent disparity in the prevalence of injury shown in Indigenous and non-Indigenous populations worldwide has been questioned. It has been suggested that mainstream injury prevention strategies are ineffective within Indigenous communities [5]. Evidence suggests that mainstream injury prevention programs, which are often urban-based, fail to meet the needs of communities living in rural environments [15], like the many Indigenous communities who maintain a traditional lifestyle in rural areas. Limitations of mainstream injury prevention strategies are thought to be too rigid and insufficiently take into account the local conditions, culture, social structures and the limited resources available to Indigenous service providers [5]. It has been suggested that intervention strategies need to go beyond the traditional approaches and tap into ethnographic and other qualitative research paths in order to better evaluate the historical differences, social structures and cultures of Indigenous populations [5]. Despite this theory, there is limited knowledge of what types of injury prevention strategies tailored for Indigenous children are effective [12,16]. The main aim of this review is identify and describe the main characteristics of existing interventions in the last 20 years which have addressed child injury prevention in Indigenous populations in Canada, Australia and New Zealand. To the best of the authors’ knowledge, no prior systematic reviews exist looking at interventions specifically aimed at preventing injury in Indigenous child populations in these countries.

## 2. Materials and Methods

The review was completed in accordance to the Preferred Reporting Items for Systematic Reviews and Meta-Analyses (PRISMA) guidelines [17]. Literature searches were conducted in Medline, CINAHL, AMED and the Child Development & Adolescent Studies. These databases were systematically searched for relevant interventions based in Canada, Australia and New Zealand from 1996 to 2016 on 11 August 2016. The search strategy of the review consisted of three elements: population, intervention and outcome. Under the population element, the following search terms were used: “Aboriginal”, “Indigenous”, “Native”, “Tribe”, “First Nation”, “Metis”, “Inuit”, “Koori”, “Maori”, “Torres Strait”, “Child”, “Youth”, “Adolescents”, “Teen”, “Infant” and “Pediatric”. For intervention, the following search terms were used: “Intervention”, “Evaluation”, “Prevention”, “Program” and “Project”. For the outcome element, the following search terms used were: “Injury”, “Poison”, “Accident”, “Wound”, “Burn”, “Collision”, “Drowning” and “Fall”.

A manual search of the reference lists of relevant articles and a manual search of relevant websites were completed. For the website search, key terms were entered into a Google search engine, for example: “Australia government”, “Australia health departments”, “Australian Indigenous organizations”, “Australian injury prevention” and others. After a website was searched, a “snowballing” method was used. If available, the resource section of the website was visited which often led to identifying new relevant websites of other key organizations. This method was completed specifically for each country included in this review, in addition to a few notable global websites. All records were exported into *RefWorks*, an online bibliographic management program. After duplicates were removed, papers were then organized into three folders: (1) potentially relevant studies; (2) studies useful for background information and (3) irrelevant studies.

Studies were included in the review if they met the following criteria: (1) Studies of interventions aimed at the prevention of any type of childhood injury in Indigenous populations; (2) Studies based on Canadian, Australian or New Zealand Indigenous populations; (3) Interventions aimed at Indigenous children aged 0–14 years old; (4) Studies with reported outcomes; (5) All types of study designs; (6) Studies published within the last 20 years (1996–2016) and (7) Studies published in the English language. Studies which did not report specific outcomes or did not meet all of the inclusion criteria were excluded from the review. Eligible papers then underwent a quality appraisal using adapted assessment checklists [18,19]. The following key information was extracted: where the record was found; ages, gender, country, target population, sample size, aim, date of publication, types of injuries addressed, duration of intervention, evaluation design type, intervention activities, outcomes, success factors and barriers. Given the substantial heterogeneity across the studies, a meta-analysis was not appropriate but rather, a textual narrative synthesis was chosen as the most relevant and appropriate method of data synthesis for the review.

## 3. Results

**Figure 1:**
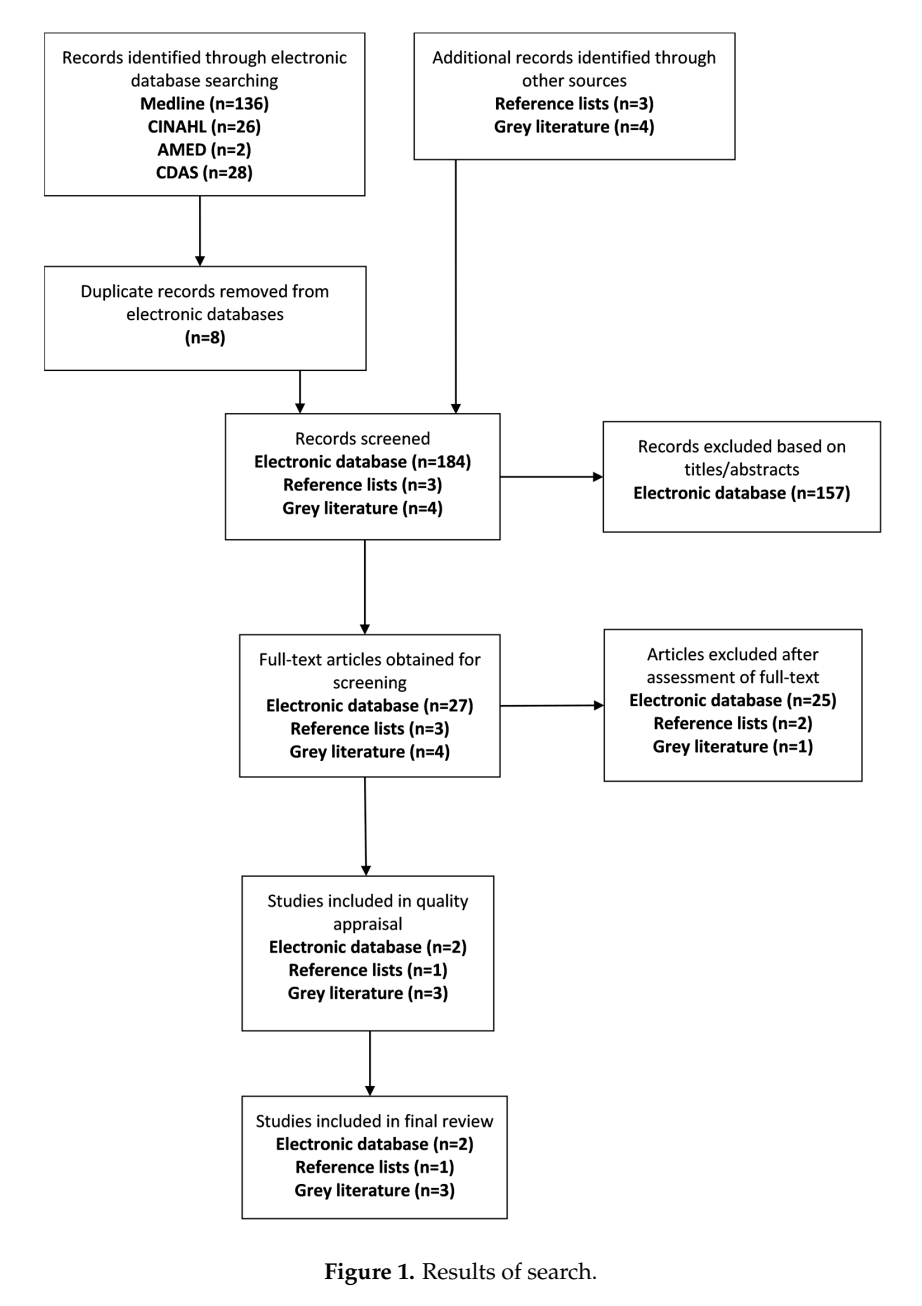
Results of search.

A total of 192 citations were identified through the electronic search procedure and seven citations were further identified through the grey literature and reference list search. After eight duplicates were removed from the electronic database search, a total of 191 citations were screened for potential eligibility. A total of 157 papers were excluded based on the screening of titles and abstracts, and a further 28 papers were removed after the assessment of the full-text. Six studies met the criteria for inclusion in the systematic review; three based in Australia and three based in New Zealand. Despite having the largest Indigenous population of the three countries, no interventions meeting eligibility criteria were found in Canada. Four of the included studies are publications based in various peer-reviewed journals while the remaining two papers, one being a full evaluation report, are published by the Australian Health Services Research Institute. Figure 1 illustrates the process to reach the final results of included papers in the current review.

### 3.1. Overview of Study Characteristics

The setting of the intervention varied across the studies; four out of six of the interventions included in the review were implemented in the community while one was implemented directly into the school-curriculum and another was implemented in an early learning center. While all studies targeted Indigenous populations; exclusive inclusion of Indigenous children and their families was not possible in many cases due to the type of intervention implemented (i.e., community-based). In these cases, study populations were chosen based on areas of high concentration of identified Indigenous families. All the community-based interventions delivered the intervention activities to all ages across the community. Activities and outcomes addressing child injuries exclusively are only described in this review.

In terms of evaluating the intervention, a mix of quantitative and qualitative methods were used, such as analyzing hospital statistics, the use of pre/post-test surveys/questionnaires, observation and interviews. Out of the four implemented in the community, one intervention was a community referral program while the other three interventions were delivered to the entire community. Child injury reduction was measured directly through the use of hospitalization statistics; three out of six of the studies included injury hospitalization rates as part of their measured outcomes; and indirectly through different proxies; the remaining three studies measured child injury prevention through one or more measures of awareness, self-efficacy and attitudes towards child injury, child restraint use, child home safety device use and parental involvement in child safety. Measures of cost effectiveness were not reported for any of the included studies.

Although a control group was not defined in the search strategy or required in the inclusion criteria, the three studies which measured child injury hospitalization rates all included a comparison community as a control. Additionally, one study used a cluster randomized design with a matched control group to measure their chosen outcome.

Only three of the six studies measured changes in injury hospitalization rates, two of these studies reporting a significant decrease. An increase in awareness of injury prevention was found across all studies measuring this outcome. Those studies which included car restraint initiatives demonstrated the most positive changes post-intervention in terms of this measure, although this was not always significant. A summary of included studies is shown in Table 1.

### 3.2. Content of Interventions

There were no studies specifically addressing one particular type of injury through their intervention. However, in terms of injury prevention areas, four out of six of included interventions focused on road safety. Three interventions addressed road safety among other areas, while another intervention addressed child car restraint use exclusively. The components of the included interventions are shown in Table 2. All intervention components included an element of education and awareness and were noted as being designed and delivered in a culturally appropriate manner. Furthermore, it is noted that the evaluation of the interventions themselves were completed based on traditional preference.

While reducing child injury was the overarching aim of all studies, many of the interventions did not target or involve children specifically, but rather, they involved the parents, educators and other organizations in the community. As children often rely heavily on their parents and other adults in their life, this approach is logical. This also highlights the holistic style of the interventions in this review; demonstrating how elements of both the individual and the environment have been considered, an underpinning of Indigenous cultures.

The inclusion of parental training sessions and teacher training workshops were components of several of the interventions. As parents are solely responsible to ensure their children are properly restrained in an age-appropriate car seat, a parent training session or workshop was commonly paired in those interventions incorporating a child car restraint component. Some interventions specifically focused on targeting parents, such as in the *Safe Homes*, *Safe Kids* program; the main intervention activities in this program directly targeted families with new babies, first time parents or teenage parents, considered at-risk. This intervention was the most concentrated in terms of following a specific model where the focus was on parental education and training as a means to address child injury in the home. Likewise, teacher education was highlighted as being equally important when addressing child injury prevention. As teachers have considerable contact with children daily, their knowledge of safety in the Indigenous culture is undoubtedly associated with a child’s learning capacity in a school environment. The incorporation of teacher training workshops was demonstrated in the *Safe Koori Kids*, *Buckle-Up Safely Shoalhaven* and *Safe Homes*, *Safe Kids* interventions.

Furthermore, a few interventions also involved community-based organizations. For example, the *Ngati Porou Community Prevention Project* involved 12 community sport and recreational clubs with the intention to address and promote safer alcohol use in these environments as part of their intervention. Likewise, the *Waitakere Community Injury Prevention Project* also assessed any changes in safety policies and practices in organizations within the community as part of this evaluation, although this was not a specific intervention activity. The inclusion of community-based organizations in the interventions highlights the importance placed on partnerships and again demonstrates a holistic approach to Indigenous child injury prevention.

### 3.3. Outcome Measures

A variety of outcomes were measured to assess intervention effectiveness; these outcomes are shown in Table 3. The evaluations of these outcomes were measured through the use of various designs; four study evaluations used a quasi-experimental design, one study used a cluster randomized design and another used a qualitative approach.

An outcome of particular interest in the current review is injury hospitalization rates. While three out of the six interventions measured this; only the *Waitakere Community Injury Prevention Project* reported a significant decrease post-intervention between the intervention community, comparison community and the rest of the region. Whereas the rates for the comparison community and rest of the region increased during the two years post intervention, hospitalization rates for the intervention community decreased. The *Turanganui-a-kiwa Community Injury Prevention Project* illustrated insignificant decreases between intervention and comparison community, while the *Ngati Porou Community Injury Prevention Project* demonstrated no differences.

Several studies measured safety knowledge and awareness; all found significant increases in these measures after intervention implementation, for children, parents and organizations within the community. Complementing the knowledge and awareness outcome is the measure of self-efficacy in adopting learned safety practices. The *Safe Koori Kids* intervention was the only intervention to quantify self-efficacy; it was found that the improved scores of the Indigenous children were greater than the improved scores of non-Indigenous children. This outcome is particularly noteworthy in the current review as it is the only measure to compare an outcome for Indigenous and non-Indigenous children separately, exclusively using statistical methods.

As previously mentioned, road safety was a main focus of many of the included interventions; the promotion of the use of appropriate car restraint use was shown across four studies. Among these, all showed an increase of child car restraint use through observational methods and pre/post-test surveys, although these findings were not always significant. Both the *Turanganui-a-kiwa Community Injury Prevention Project* and *Waitakere Community Injury Prevention Project* demonstrated significant improvements post-intervention; a 64% and 7% increase in correct car restraint use were shown post-intervention, respectively. The *Buckle-Up Safely Shoalhaven* and *Ngati Porou Community Injury Prevention Project* did not show significant differences; however, both had relatively high baseline rates pre-intervention. Out of the four studies promoting correct child car restraint use, three offered restraints by means of a loan or through a subsidized plan; these evaluations reported that the demand for restraints increased in those interventions offering this service. Moreover, resulting from the support of organizations and the increased demand for car restraints, the *Turanganui-a-kiwa Community Injury Prevention Project* reports that a Maori based car restraint loan shop was opened in the community. The *Safe Kids, Safe Homes* program was the only program to measure the use of safety devices in the home environment and found that families continued to use the devices appropriately at a three month assessment. Families expressed that without the intervention they would not have known how these devices worked. Those who wished to purchase devices prior to intervention expressed not knowing what to buy.

The two studies by Clapham et al. [10,24] both looked at attitudes towards safety. No differences were shown in a child’s attitudes towards safety in the *Safe Koori Kids* intervention. Parents in the *Safe Homes, Safe Kids* program reported a change in their attitudes after the program, expressing participation was a big “eye opener” to an important issue. It was shown that parents were more involved and engaged in community injury prevention after participation in this program. Similarly, parental involvement was shown to increase slightly in the *Safe Koori Kids* program. These are important outcomes to consider when choosing intervention activities promoting the prevention of child injuries, and whom they should relate to the most.

### 3.4. Success Factors

In terms of critical success factors, there were several themes which were identified across all interventions. One factor noted by all studies as affording success to the intervention was the culturally appropriate content of the intervention. Interventions were designed in a way where their framework was underpinned by local traditions and customs. Coggan et al. [22] note that community members of Ngati Porou felt that the project belonged to them and because the program was seen as being culturally relevant, this therefore increased the acceptability and active participation in the program. The design of the intervention in a culturally appropriate manner was made possible through the inclusion of Indigenous service providers and community members in the design and implementation. Hunter et al. [23] report collaborating with a local advisory committee comprising of researchers of Aboriginal descent to acquire advice on the best way to conduct their research prior to the commencement of their pilot study. Similarly, Clapham et al. [10] note that an Aboriginal research advisory committee was created specifically for the *Safe Koori Kids* project to help with the direction of strategies. Actively involving community gate keepers is also noted as an important part of the design and planning stage. Coggan et al. [22] report that as the intervention was developed using a Maori framework, the team was able to successfully draw together many Indigenous groups and organizations to work together toward injury prevention.

Furthermore, all studies note involvement directly in the delivery of the intervention by persons of Indigenous descent and/or persons knowledgeable of the Indigenous culture specific to that area. This type of involvement was highlighted by all studies as a main strength of the intervention. For example, as Aboriginal families had expressed having previous negative experiences with mainstream services lacking contact with Aboriginal workers who were familiar and knowledgeable of the complex needs of this population, the use of Aboriginal Family Workers in the *Safe Homes, Safe Kids* program was noted as a main success of their intervention and evaluated positively by participants. This allowed a more trusting relationship to be built with families, enabling successful delivery and improved the effectiveness of the program. The positive impacts of home visiting programs and the use of professional home visitors has been supported in the literature [11].

A holistic approach, aligning with Indigenous traditional practices, is one that addresses health and well-being in terms of physical, mental, emotional and spiritual aspects of life, was also noted as an integral factor across all interventions. The interventions delivered in a holistic way, recognized that health is socially determined and operates under this principle. Clapham et al. [24] attribute success of the program to the holistic health service delivery used, allowing the opportunity for internal and external referrals to a wide range of services to be made possible.

Providing access to subsidized or free safety devices is also reported as a success factor in the interventions involving this. The uptake of child restraints was significantly increased in those interventions providing access to these on a subsidized loan scheme, although the *Buckle-Up Safely Shoalhaven* intervention does report that several families still chose not participate in the subsidized restraint scheme due to financial hardships. Families participating in the *Safe Homes, Safe Kids* program expressed the view that they would not have invested in home safety devices themselves as they were unable to afford them. This demonstrates how financial restrictions place a barrier to the uptake of safety devices but funding support in these areas can facilitate the prevention of child injury.

### 3.5. Barriers

In terms of barriers, there were a few noted as hindering the program effectiveness and evaluation. One in particular was the issue around staff turnover leading to a lack of cohesion and consistency throughout intervention. Clapham et al. [24] note that a main challenge in the *Safe Homes, Safe Kids* program was retaining experienced staff. Additionally, there was a limited availability of experienced Aboriginal workers to employ as replacements. Similarly, it was felt that results of the *Buckle-Up Safely Shoalhaven* intervention were adversely affected by the turnover of all staff at one of the early learning centers, negatively affecting the promotion of parent training sessions and delayed uptake of child restraints. These types of barriers shown in these interventions and their evaluations act as an important reminder of the real challenges faced in these situations.

Another barrier of note, observed in the *Safe Home, Safe Kids* program found some families were unwilling to allow Aboriginal workers into their home. The authors speculate that this could have been due to families fearing workers would report suspected child abuse. As this study was the only intervention involving a home visiting component, no other studies reported a barrier such as this. The positive findings of this home visiting program were encouraging but this reported barrier demonstrates the need for further evaluations assessing the potential obstacles involved in home visiting interventions.

## 4. Discussion

In accordance with the inclusion/exclusion criteria, the six included interventions in the review are aimed at the reduction of injury in Indigenous children. However, they varied in terms of the study design, setting, target population, intervention type and outcome measures; thus presenting methodological heterogeneity. The presence of methodological variability limits the generalizability of review findings. However, despite this variability, the reasons behind positive findings were similar, thus the parallel themes across the interventions demonstrating a degree of generalizability. Overall findings confirm that there is a limited amount of high quality evidence addressing injury prevention for Indigenous children. Quality of evidence was affected largely by the lack of methodological rigor across several of the included studies, presenting a main limitation in this review.

The first issue relating to the methodological rigor is the incomplete description of the content of the intervention activities. A lack of a comprehensive description of interventions published in journals hindered by a journal’s word allowance has been highlighted in the literature as an issue [25]. In a study measuring completeness, over half of the published studies assessed failed to provide a sufficient description of the intervention process to allow for replication [25]. This issue was also experienced during the current review process; due to a complete absence of a description of intervention components and outcome measurements, one study was excluded during the eligibility assessment stage for this reason. The evaluation of the *Safe Homes, Safe Kids* program was not a peer-reviewed publication but rather was a full-text evaluation report and thus was able to provide a comprehensive amount of valuable information obtained from qualitative interviews of the experiences and views of Aboriginal families which took part in the program. This evaluation was assessed highly in terms of quality, clarity and comprehensiveness during the quality appraisal stage. It is recognized that a rigorous evaluation such as this one is undoubtedly difficult to execute without the appropriate resources in place. This particular program is well-established and had been operating for a period of 10 years prior to its evaluation which likely reflects the capacity to fund an in-depth evaluation. Efforts to sustain programs such as this one can be challenging due to the limited capacity of these organizations and insufficient funding [11,26] and it is not difficult to recognize that small scale interventions lacking the resources to (a) grow in size and reach, and (b) fund a rigorous evaluation, consequently affect the intervention’s ability to become translated into a larger strategy or policy. Given the overwhelming amount of existing literature describing high child injury rates in Indigenous populations, but lacking evidence on what works, and how it works, a detailed description of injury prevention interventions is essential in order to build the evidence base and to inform researchers, policy-makers and those seeking to develop culturally appropriate interventions.

A second issue relating to the methodological rigor is the length of the interventions. Five out of six of the studies were implemented as short pilot projects and none assessed any long-term impacts on the community. Longitudinal interventions in addition to long-term follow-up are ways to improve the evidence base in this area.

A third issue relating to the methodological rigor is the potential bias in measures. The analysis of child hospitalization rates and mortality due to injury is an objective method to measure injury reduction, but, only three of the included interventions chose to analyze hospitalization rates and none chose to analyze mortality due to injury. Several reasons for this included the expectation that: the intervention would not result in immediate impacts on rates, the likelihood to detect any differences in such a small population was improbable and given the short duration of the intervention and limited resources, and analyzing rates was unrealistic. Consequently, the reliance of self-reported measures or reports from key informants to assess outcomes was common across the studies. The included studies relied on a small sample of a non-random selection of self-reported data, reducing the reliability of this outcome. The use of self-reported survey data in public health practice is desirable because it is an economic way to analyze intervention effects [27], but self-reported measures which are used in a pre/post-test approach have the potential to be confounded by a response shift as participants may lean toward providing favorable evaluations of the given intervention [28]. However, this method of data collection has shown potential to provide a valuable source of information. Clapham et al. [24] note that the length of time required for hospitalization rates to become available to incorporate in their study was too long. Therefore, they asked parents to provide examples of where injuries had been prevented because of participation in the *Safe Homes, Safe Kids* program. While the analysis of long-term hospitalization rates of child injuries can act as a credible measurement tool and has the potential to minimize concerns of self-reported bias, flaws and other biases in utilizing hospital data remain present [29]. The findings of this review reveal that limitations exist in respect to the use of hospital injury data, particularly surrounding the issue of accuracy in terms (1) the completeness of all injuries and (2) ethnicity identifiers. 

The integrity of hospital injury data can be affected by the inability for this data to capture less medically serious injuries [30]. Children sustaining these injuries would likely not present at the hospital, resulting in the burden of injury being underestimated [11,31]. Thus, the utilization of parental self-reports of specific examples can provide valuable information about these less serious injuries in a more rapid manner and may assess the injury burden more appropriately. Moreover, a greater issue in terms of Indigenous research is the lack of recording, or incorrect recording, of ethnicity identifiers in hospital data [30], which does not allow for correct estimates of child injury rates to be made. Once more, this can result in the burden of injury for Indigenous populations being underestimated, resulting in serious implications for overall Indigenous health. From a public health perspective, there are potential dangers associated with inaccurate or incomplete ethnicity recording. Apparent declines could lead to injuries or other health issues no longer being viewed as problematic and less prioritization would be given to the area resulting in complacency in respect to prevention strategies [4]. When addressing health disparities; inferences made from hospital statistics for any health outcome, not just injury, must be done with caution.

In terms of weaknesses in the review process itself, one main weakness is the possibility that interventions based in the grey literature were missed. During the grey literature search, several on-line websites mentioned programs or strategies, but formal evaluations of these interventions were not found. It is possible that other interventions exist without any published formal evaluations, and therefore would not be included in this review. This issue has been documented in the literature to be common among injury prevention research [12,32]. This phenomenon was likewise observed during this review, illustrated in the fact that only two out of the six included studies were found through the electronic database search. Avoiding publication bias is challenging as there is always a possibility of the presence of this bias in reviews [32]. Reaching out to other potential sources can help minimize this bias. Although time and resources for this current review affected the ability to reach out directly to Indigenous leaders and communities who may have unpublished findings, this strategy could allow for the study to be expanded in the future by providing additional sources of information. Nonetheless, great efforts were made to identify all possible studies but publication bias can still not be ruled out in this review. Additionally, English papers were only included in this review, presenting a language bias and the possibility that other studies published in a language other than English were missed.

## 5. Conclusions

The findings of this review confirmed that evaluated interventions aimed at the prevention of Indigenous child injuries are limited. Findings suggest that culturally appropriate interventions and involvement of Indigenous communities in the design and delivery of interventions resulted in changes in knowledge and awareness of child injuries. The inclusion of Indigenous communities in shaping interventions and further policies is not only important in terms of intervention effectiveness, but it underpins the principles of Indigenous rights to self-determination and cultural preservation.

Evidence of effective interventions aimed at the prevention of childhood injuries in Indigenous populations was encouraging but not conclusive. This was mainly due to the challenges associated with drawing inferences from limited amount of variable, small-scale evaluations of pilot interventions lacking methodological rigor. Furthermore, evidence was not conclusive whether programs designed specifically for Indigenous children have a greater ability to reduce child injury rates and are of a greater benefit than mainstream injury prevention programs.

In order to strengthen the evidence-base in this area in the future, improvements in the following areas are recommended as follows:

- Increase the number of published evaluations with comprehensive descriptions of the interventions.
- Improve the reliability of measures within intervention evaluations by improving self-reported measurement tools, or moving towards more rigorous measurement tools.
- Increase the number of longitudinal interventions, combined with evaluations analyzing the effects over time, specific to the Indigenous population.
- Capture injury statistics more accurately and drive the need for improvements in ethnicity recording in hospital datasets.
- Further address the issue surrounding unevaluated, unpublished interventions.
- Design, evaluate and publish up-to-date interventions addressing the present-day needs of Indigenous populations.
- Compare tailored versus mainstream interventions in same communities.

The findings in this review are not only relevant to preventing child injuries, but rather provide insight on ways to improve overall health of Indigenous children. The findings act as a reminder of the complexity of Indigenous health which future research must bear in mind. Centuries of past injustices, disenfranchisement and oppression are at the roots of the social determinants of the poor health in these populations. Interventions must be underpinned by a critical understanding of the greater effects of poverty and marginalization of Indigenous people within their countries.

## Figures and Tables

**Table 1:** Summary of included studies in the review.

Intervention Name	Intervention Type/ Methods of Evaluation	Target Population	Targeted Injury Type
**Safe Koori Kids Study** (Clapham et al., 2012 [10])	School-based intervention evaluated with a pre-test/post-test quasi experimental design.	Aboriginal children of primary school aged living in the Campbelltown area in Australia	All injury types
**Turanganui-a-kiwa Community Injury Prevention Project** (Brewin and Coggan, 2002 [20])	Community-based intervention evaluated with a quasi-experimental design with comparison community.	Maori Children aged 0–14 living in the Turanganui-a-kiwa community in New Zealand	Road injuries
**The Waitakere Community Injury Prevention Project** (Coggan et al., 2000 [21])	Community-based intervention evaluated with a quasi-experimental design with comparison community.	Maori children aged 0–14 living in Waitakere Community in New Zealand	All injury types
**Ngati Porou Injury Prevention Project** (Brewin and Coggan, 2004 [22])	Community-based intervention evaluated with a quasi-experimental design with comparison community.	Maori children living in Ngati Porou Community in New Zealand	All injury types
**Buckle-up Safely: Shoalhaven** (Hunter et al., 2014 [23])	School-based intervention evaluated using a cluster randomized controlled design.	Aboriginal children aged 3–5 years old attending three early-learning centers in the Shoalhaven community in Australia	Road injuries
**Safe Homes, Safe Kids Program** (Clapham et al., 2015 [24])	Community-based home visiting referral program evaluated using a mixed methods design.	Families with young children aged 0–5 years old living in the Illawara region in Australia	All injury types with a focus on injuries having the potential to occur inside the home

**Table 2:** Intervention components of included studies in the review.

Intervention Name	Home Safety Assessment	Interactive Game	Educational Material	Teacher Training Workshop	Media Campaign	Training Sessions on Safety Practices	Car Restraint Loan/Subsidization	Bus Monitoring Program	Organizational Workshops	Parental Workshops
Safe Koori Kids Study [10]		X	X	X						X
Turanganui-a-kiwa Community Injury Prevention Project [20]					X	X	X			
The Waitakere Community Injury Prevention Project [21]			X						X	
Ngati Porou Injury Prevention Project [22]			X			X	X	X		
Buckle-up Safely: Shoalhaven [23]			X	X			X			X
Safe Homes, Safe Kids Program [24]	X			X		X				

**Table 3:** Measured intervention outcomes of included studies in the review.

Intervention Name	Injury Hospitalization Rates	Safety Knowledge and Awareness	Self-Efficacy in Adopting Safety Behaviors	Child Car Restraint Use	Home Child Safety Device Use	Attitudes toward Safety	Parental Involvement in Community Safety
rigoSafe Koori Kids Study [10]		X	X			X	X
Turanganui-a-kiwa Community Injury Prevention Project [20]	X			X			
The Waitakere Community Injury Prevention Project [21]	X	X		X			
Ngati Porou Injury Prevention Project [22]	X	X		X			
Buckle-up Safely: Shoalhaven [23]				X			
Safe Homes, Safe Kids Program [24]		X			X	X	X

## References

[1] Cooke M., Mitrou F., Lawrence D., Guimond E., Beavon D. (2007). Indigenous well-being in four countries: An application of the UNDP’S human development index to Indigenous peoples in Australia, Canada, New Zealand, and the United States. BMC Int. Health Hum. Rights.

[2] Jahan S. (2016). Human Development Report 2016.

[3] Murray C.J., Lopez A.D. (1997). Alternative projections of mortality and disability by cause 1990–2020: Global Burden of Disease Study. Lancet.

[4] George M.A., Jin A., Brussoni M., Lalonde C.E., McCormick R. (2015). Injury risk in British Columbia, Canada, 1986 to 2009: Are Aboriginal children and youth over-represented?. Inj. Epidemiol..

[5] Ivers R., Clapham K., Senserrick T., Lyford M., Stevenson M. (2008). Injury prevention in Australian Indigenous communities. Injury.

[6] Australian Bureau of Statistics 3303.0-Causes of Death, Australia, 2013: Leading Causes of Aboriginal and Torres Strait Islander Deaths. http://www.abs.gov.au/ausstats/abs@.nsf/Lookup/by%20Subject/3303.0~2013~Main%20Features~Leading%20Causes%20of%20Aboriginal%20and%20Torres%20Strait%20Islander%20Deaths~10015.

[7] Postl B., Cook C., Moffatt M. (2010). Aboriginal child health and the social determinants. Healthc. Q..

[8] Hodges I., Maskill C., Coulson J., Christie S., Quigley R. (1998). Our Children’s Health: Key Findings on the Health of New Zealand Children.

[9] Moller H., Falster K., Ivers R., Jorm L. (2015). Inequalities in unintentional injuries between Indigenous and non-Indigenous children: A systematic review. Inj. Prev..

[10] Clapham K.F., Khavarpour F., Bolt R.J., Stevenson M., Su S. (2012). Researching the safety of Indigenous children and youth: An urban perspective. Urban Health: Strengthening Our Voice, Culture and Partnerships, Proceedings of the 2009 AIATSIS National Indigenous Studies Conference, Canberra, Australian Capital Territory, Australia, 29 September–1 October 2009.

[11] Peden M. (2008). World Report on Child Injury Prevention.

[12] Oliver L.N., Kohen D.E. (2012). Unintentional injury hospitalizations among children and youth in areas with a high percentage of Aboriginal identity residents: 2001/2002 to 2005/2006. Health Rep..

[13] Brussoni M., Jin A., George M.A., Lalonde C.E. (2015). Aboriginal community-level predictors of injury-related hospitalizations in British Columbia, Canada. Prev. Sci..

[14] Pulver L.J., Haswell M.R., Ring I., Waldon J., Clark W., Whetung V., Kinnon D., Graham C., Chino M., LaValley J. (2010). Indigenous Health–Australia, Canada, Aotearoa New Zealand and the United States-Laying Claim to a Future That Embraces Health for Us All.

[15] Smith K.B., Humphreys J.S., Wilson M.G. (2008). Addressing the health disadvantage of rural populations: How does epidemiological evidence inform rural health policies and research?. Aust. J. Rural Health.

[16] Moller J., Thomson N., Brooks J. (2004). Injury Prevention Activity among Aboriginal and Torres Strait Islander Peoples. Volume 1: Current Status and Future Directions.

[17] Moher D., Liberati A., Tetzlaff J., Altman D.G., Prisma Group (2009). Preferred reporting items for systematic reviews and meta-analyses: The PRISMA statement. PLoS Med..

[18] National Institute for Health and Clinical Excellence (2012). Methods for the Development of NICE Public Health Guidance (Third Edition): Appendix H Quality Appraisal Checklist—Qualitative Studies.

[19] National Institute for Health and Clinical Excellence (2012). Methods for the Development of NICE Public Health Guidance (Third Edition): Appendix F Quality Appraisal Checklist—Quantitative Studies.

[20] Brewin M., Coggan C. (2002). Evaluation of a New Zealand Indigenous community injury prevention project. Inj. Control Saf. Promot..

[21] Coggan C., Patterson P., Brewin M., Hooper R., Robinson E. (2000). Evaluation of the Waitakere Community Injury Prevention Project. Inj. Prev..

[22] Brewin M., Coggan C. (2004). Evaluation of the Ngati Porou community injury prevention project. Ethn. Health.

[23] Hunter K., Keay L., Clapham K., Lyford M., Brown J., Bilston L., Simpson J.M., Stevenson M., Ivers R.Q. (2014). Buckle up safely (Shoalhaven): A process and impact evaluation of a pragmatic, multifaceted preschool-based pilot program to increase correct use of age-appropriate child restraints. Traffic Inj. Prev..

[24] Clapham K.F., Bennett-Brook K.R., Callister C.P., Fildes D.L. (2015). Evaluation of the Illawarra Aboriginal Medical Service ‘Safe Homes, Safe Kids’ Program.

[25] Douet L., Milne R., Anstee S., Habens F., Young A., Wright D. (2014). The completeness of intervention descriptions in published National Institute of Health Research HTA-funded trials: A cross-sectional study. BMJ Open.

[26] Mitchell R. (2014). Data linkage for paediatric trauma and health services research. Report from the Paediatric Injury Management Research Forum, Proceedings of the Paediatric Injury Management Research Forum, Sydney, Australia, 1 August 2014.

[27] Bauhoff S. (2011). Systematic self-report bias in health data: Impact on estimating cross-sectional and treatment effects. Health Serv. Outcomes Res. Methodol..

[28] Howard G.S., Ralph K.M., Gulanick N.A., Maxwell S.E., Nance D.W., Gerber S.K. (1979). Internal invalidity in pretest-posttest self-report evaluations and a re-evaluation of retrospective pretests. Appl. Psychol. Meas..

[29] Towner E., Dowswell T. (2002). Community-based childhood injury prevention interventions: What works?. Health Promot. Int..

[30] Australian Institute of Health and Welfare (2016). Australia’s Health 2016.

[31] Thompson S.C., Woods J.A., Katzenellenbogen J.M. (2012). The quality of Indigenous identification in administrative health data in Australia: Insights from studies using data linkage. BMC Med. Inform. Decis. Mak..

[32] Blackhall K. (2007). Finding studies for inclusion in systematic reviews of interventions for injury prevention the importance of grey and unpublished literature. Inj. Prev..

